# Implementation of Remote Interventions in Older Adults: Lessons Learned During COVID-19 Isolation

**DOI:** 10.1093/geroni/igaa057.3476

**Published:** 2020-12-16

**Authors:** Jamie Rincker, Jessica Wallis, Angela Fruik, Alyssa King, Kenlyn Young, Thoma Tucker, Connie Bales, Kathryn Porter Starr

**Affiliations:** 1 Duke University, Durham, North Carolina, United States; 2 Duke University School of Medicine, Durham, North Carolina, United States

## Abstract

Recommendations for older adults to socially isolate during the COVID-19 pandemic will have lasting impacts on body weight and physical activity. Due to the pandemic, two in-person RCT weight-loss interventions in obese older adults with prediabetes, Veterans Achieving Weight Loss and Optimizing Resilience-Using Protein (VALOR-UP, n=12) and the Egg-Supplemented Pre-Diabetes Intervention Trial (EGGSPDITE, n=7), were converted to remote formats and weekly nutrition (EGGSPDITE and VALOR-UP) and exercise (VALOR-UP only) classes were delivered using synchronous videoconference technology (Webex); classes were accessed via tablet/desktop/laptop or smart phone. Steps taken to transition participants to remote formats included technology training, implementation of staff tech-support, and delivery of nutrition education, tablets, scales, and exercise bands. The time to successfully transition participants was 1 week for early adopters (n=10) and up to 4 weeks for those with significant technology barriers (n=9); their difficulties included internet access, camera and microphone access and use, and electronic submission of weight and food records. Even with these challenges, in the first 3 months of remote delivery, participant dropout rate was low (10.5%, n=2), attendance was high (87.6% nutrition class (n=19); 76.4% exercise class (VALOR-UP, n=12)), and weight loss was successful (>2.5% loss (n=13); >5% loss (n=8)), showing that lifestyle interventions can be successfully adapted for remote delivery. Remote interventions also have potential for use in non-pandemic times to reach underserved populations who often have high drop-out rates due to caretaker roles, transportation limitations, and work schedules. These barriers were significantly reduced using a virtual intervention platform.

